# Exploring ALDH2 expression and immune infiltration in HNSC and its correlation of prognosis with gender or alcohol intake

**DOI:** 10.1038/s41598-022-06244-1

**Published:** 2022-02-15

**Authors:** Senbang Yao, Xiangxiang Yin, Tingting Chen, Wenjun Chen, He Zuo, Ziran Bi, Xiuqing Zhang, Yanyan Jing, Lulian Pang, Huaidong Cheng

**Affiliations:** 1 Department of Oncology, The Second Affiliated Hospital of Anhui Medical University, Anhui Medical University, Hefei, 230601 Anhui China; 2 Department of Oncology, Anhui Chest Hospital, Hefei, Anhui China

**Keywords:** Cancer, Computational biology and bioinformatics, Biomarkers, Medical research, Molecular medicine, Oncology

## Abstract

The aldehyde dehydrogenase 2 point mutation (ALDH2*2) is a common frequent human gene variant, especially in East Asians. However, the expression and mechanism of action of ALDH2 in HNSC remain unknown. The present study explored the clinical significance and immune characteristics of ALDH2 in HNSC. The receiver operating characteristic curve was analysed to assess the diagnostic value of ALDH2 expression. ALDH2 expression in normal tissues and HNSC tissues was evaluated by IHC, and we also analysed ALDH2 gene expression in 4 HNSC cell lines. ALDH2 expression was significantly reduced in HNSC tissues compared to normal tissues (*p* < 0.05). HNSC patients with high ALDH2 expression had a better prognosis compared to patients with low ALDH2 expression (*p* < 0.05). GSEA indicated that these gene sets were correlated with signalling pathways, including the JAK-STAT signalling pathway. Unexpectedly, we found a significant prognostic effect of ALDH2 for HNSC based on alcohol consumption and the male sex. The correlation between ALDH2 expression and immune inhibitors showed an effect for ALDH2 in modifying tumour immunology in HNSC, and there may be a possible mechanism by which ALDH2 regulates the functions of T cells in HNSC. In addition, we developed a prognostic nomogram for HNSC patients, which suggested that low ALDH2 expression indicated poor prognosis in HNSC patients who were males and alcoholics.

## Introduction

Head and neck squamous carcinoma (HNSC) is the eighth most common malignancy according to the information reported in Global Cancer Statistics 2021^1^. The most promising method to reduce mortality is early cancer diagnosis as early detection is correlated with a more favourable prognosis for almost all types of cancer^2^. Currently, there is a lack of accurate biomarkers for the detection of HNSC. With the development of high-throughput sequencing technology, many important diagnostic genes are constantly being discovered. However, key biomarkers that can be used to enhance the prognosis of patients with head and neck squamous cell carcinoma remain to be confirmed.

Acetaldehyde dehydrogenase 2 (ALDH2) is a main enzyme for acetaldehyde metabolism during alcohol metabolism. ALDH2 is acknowledged for its alcohol oxidation among many aldehyde dehydrogenase genes, and approximately 30% to 40% of Asians have genetic defects in this enzyme. Individual exposure to large amounts of the catalytic active form of acetaldehyde may also make the individual more susceptible to many types of cancer. ALDH2 gene defects are correlated with an increased risk of hepatocellular carcinoma in patients with hepatitis B cirrhosis due to excessive alcohol consumption^3^. Jin et al. showed that ALDH2 plays a key role as a cancer suppressor by sustaining the stability of the liver genome, and the common human ALDH2 mutation may be an important risk factor for liver cancer^4^. A recent study has found a significant positive dose–response correlation between DFS and drinking history in HNSC patients with ALDH2 Glu/Glu^5^. However, the function of ALDH2 in HNSC remains unclear.

In the present study, we explored ALDH2 expression in numerous neoplasms using The Cancer Genome Atlas (TCGA) and its association with HNSC patient prognosis. Gene set enrichment analysis (GSEA) was applied to further evaluate the biological functions of the ALDH2 regulatory network correlated with HNSC pathogenesis. Because infiltration of immune cells is vital for the prognosis of HNSC patients, we also analysed the connection between the expression of ALDH2 and the immune cell infiltration score. Our research provides new insights into the function of ALDH2 in head and neck squamous cell carcinoma.

## Results

### Patient characteristics

Gene sequence expression data of 502 HNSC cases were downloaded from TCGA website (https://portal.gdc.cancer.gov/). The clinical characteristics of the 502 HNSC cases are shown in Table 1. The age ≤ 60 group accounted for half of the sample (50.00%) followed by the age > 60 group (50.00%). There were 33 cases (6.80%) in Group T1, 144 cases (29.50%) in Group T2, 131 cases (26.90%) in Group T3 and 179 cases (36.8%) in Group T4. N0 type cases were the majority (49.80%, N = 239) followed by N1 type (16.70%) [80], N2 type (32.10%) [154] and N3 type (1.40%)^7^. The percentages of the different HNSC stages were as follows: Stage I HNSC cases accounted for 3.90%^19^; Stage II cases accounted for 19.50% [95]; Stage III cases accounted for 20.90% [102]; and Stage IV cases accounted for 55.80% [272]. Out of the 502 HNSC patients, 5 (1.0%) had distant metastasis. The sex composition of HNSC cases was 134 females and 368 males.

**Table 1:** TCGA-HNSC patient characteristics.

Characteristic	Levels	Low expression of ALDH2	High expression of ALDH2
n		251	251
Gender, n (%)	Female	63 (12.5%)	71 (14.1%)
	Male	188 (37.5%)	180 (35.9%)
Age, n (%)	< = 60	124 (24.8%)	121 (24.2%)
	> 60	127 (25.3%)	129 (25.7%)
T stage, n (%)	T1	13 (2.7%)	20 (4.1%)
	T2	63 (12.9%)	81 (16.6%)
	T3	75 (15.4%)	56 (11.5%)
	T4	90 (18.5%)	89 (18.3%)
N stage, n (%)	N0	106 (22.1%)	133 (27.7%)
	N1	38 (7.9%)	42 (8.8%)
	N2	85 (17.7%)	69 (14.4%)
	N3	6 (1.2%)	1 (0.2%)
M stage, n (%)	M0	234 (49.1%)	238 (49.9%)
	M1	2 (0.4%)	3 (0.6%)
Clinical stage, n (%)	Stage I	7 (1.4%)	12 (2.5%)
	Stage II	42 (8.6%)	53 (10.9%)
	Stage III	54 (11.1%)	48 (9.8%)
	Stage IV	138 (28.3%)	134 (27.5%)
Alcohol history, n (%)	No	70 (14.3%)	88 (17.9%)
	Yes	176 (35.8%)	157 (32%)
Race, n (%)	Asian	7 (1.4%)	3 (0.6%)
	Black or African American	29 (6%)	18 (3.7%)
	White	207 (42.7%)	221 (45.6%)
Age, median (IQR)		61 (52, 69)	61 (55, 69)

### The expression of ALDH2 at the transcriptional level and its diagnostic function

**Figure 1:**
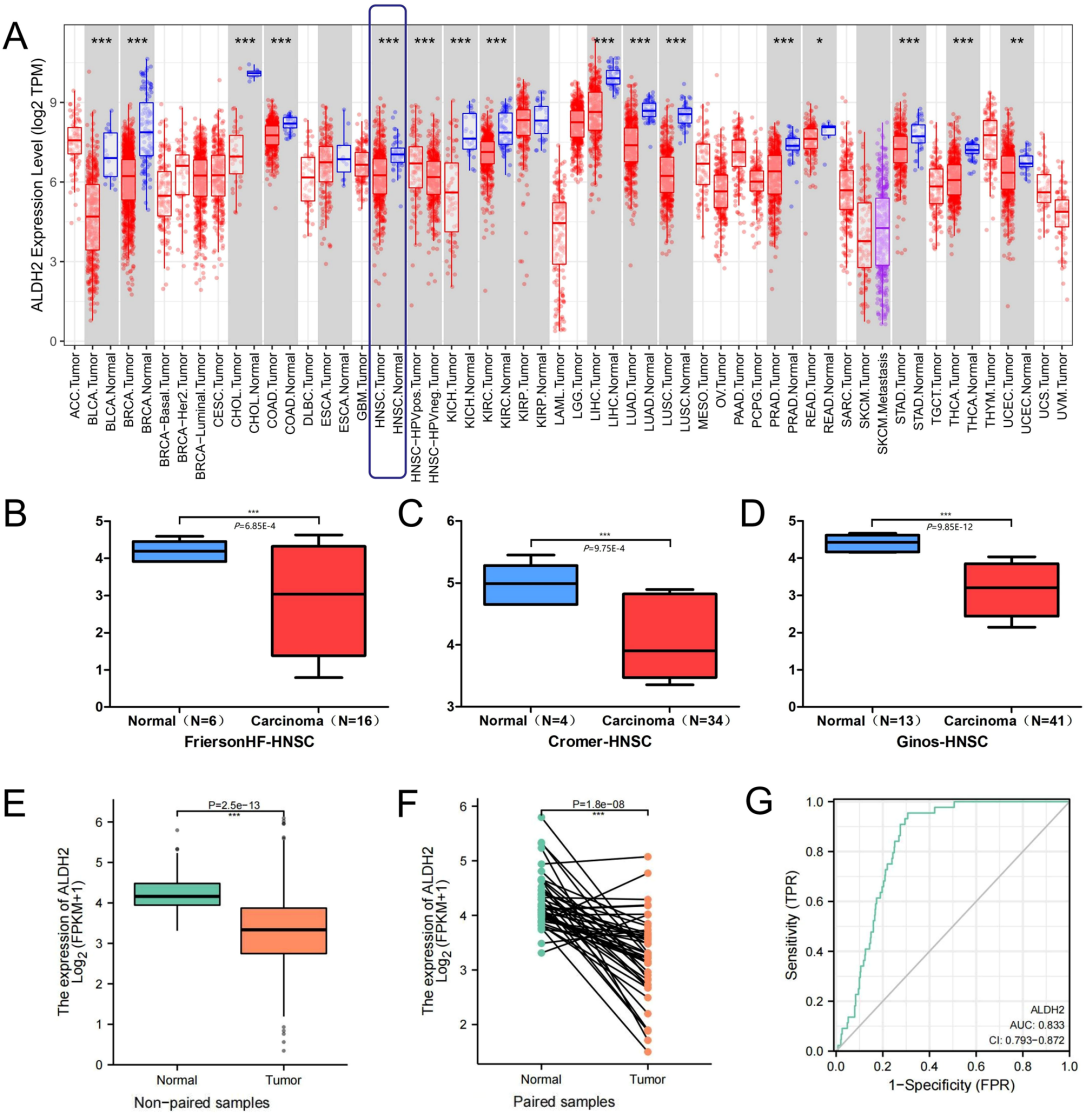
The expression of ALDH2 at the transcriptional level and its diagnostic value. (**A**) TCGA pan-cancer data showing the expression of ALDH2 in tumours and normal tissues. (**B–D**) The expression of ALDH2 was significantly reduced in 91 HNSC tissues compared to 23 normal tissues in the three datasets (FriersonHF-HNSD, Cromer-HNSC and Ginos-HNSC). (**E**) ALDH2 expression in unpaired normal and HNSC tumour tissues from TCGA-GTEx. (**F**) ALDH2 expression in paired normal and HNSC tumour tissues from TCGA. (**G**) ROC curves of ALDH2 expression in normal and HNSC tumour tissues.

The ALDH2 expression from TCGA pan-cancer data was analysed. Compared to normal tissues, the expression level of ALDH2 in cancer tissues was significantly reduced (Fig. 1A). To verify the expression of ALDH2 in HNSC patients, we used 3 related GEO datasets, which presented sequencing results with similar ALDH2 expression differences in TCGA-HNSC (Fig. 1B–D). ALDH2 expression differences between nonpaired samples were statistically significant as shown in Fig. 1E. Among 36 pairs of matched tissues, the expression of ALDH2 in tumour tissues and paraneoplastic tissues was also significantly different (Fig. 1F). To evaluate the diagnostic efficacy of ALDH2, we performed ROC curve analysis on the expression data from tumour and normal tissues. The area under the ROC curve was 0.833 [95% confidence interval (CI): 0.793–0.872] (Fig. 1G).

### Expression of ALDH2 in tissues and cell lines

**Figure 2:**
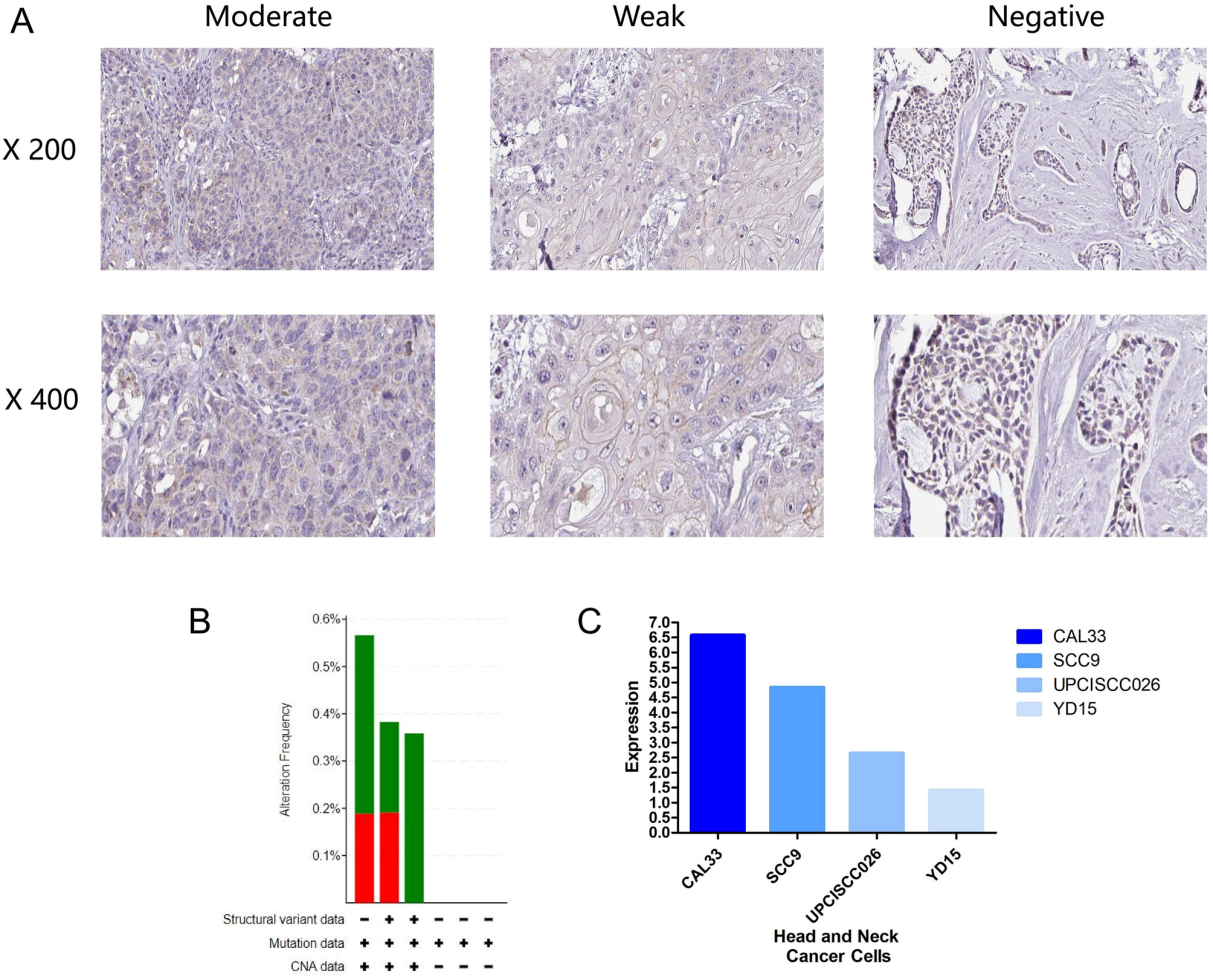
ALDH2 expression in tissues and cell lines. (**A**) ALDH2 protein expression staining (upper, × 200; lower, × 400) showing that ALDH2 protein expression was reduced in tumour tissues. (**B**) ALDH2 mutation frequencies across HNSCs obtained from cBioPortal. (**C**) Relative expression of ALDH2 in different HNSC cell lines.

Immunohistochemistry (IHC) staining showed the differential expression of ALDH2 in HNSC tissues. The staining intensity and quantity were significantly downregulated in HNSC compared to normal tissue (Fig. 2A). We also examined the ALDH2 alteration frequency across HNSCs (Fig. 2B). Importantly, HNSC cell lines had different ALDH2 expression levels with the highest expression in CAL33 HNSC cells and the lowest expression in YD15 cells lines compared to the other cell lines (Fig. 2C).

### ALDH2 expression and clinicopathological parameters

**Figure 3:**
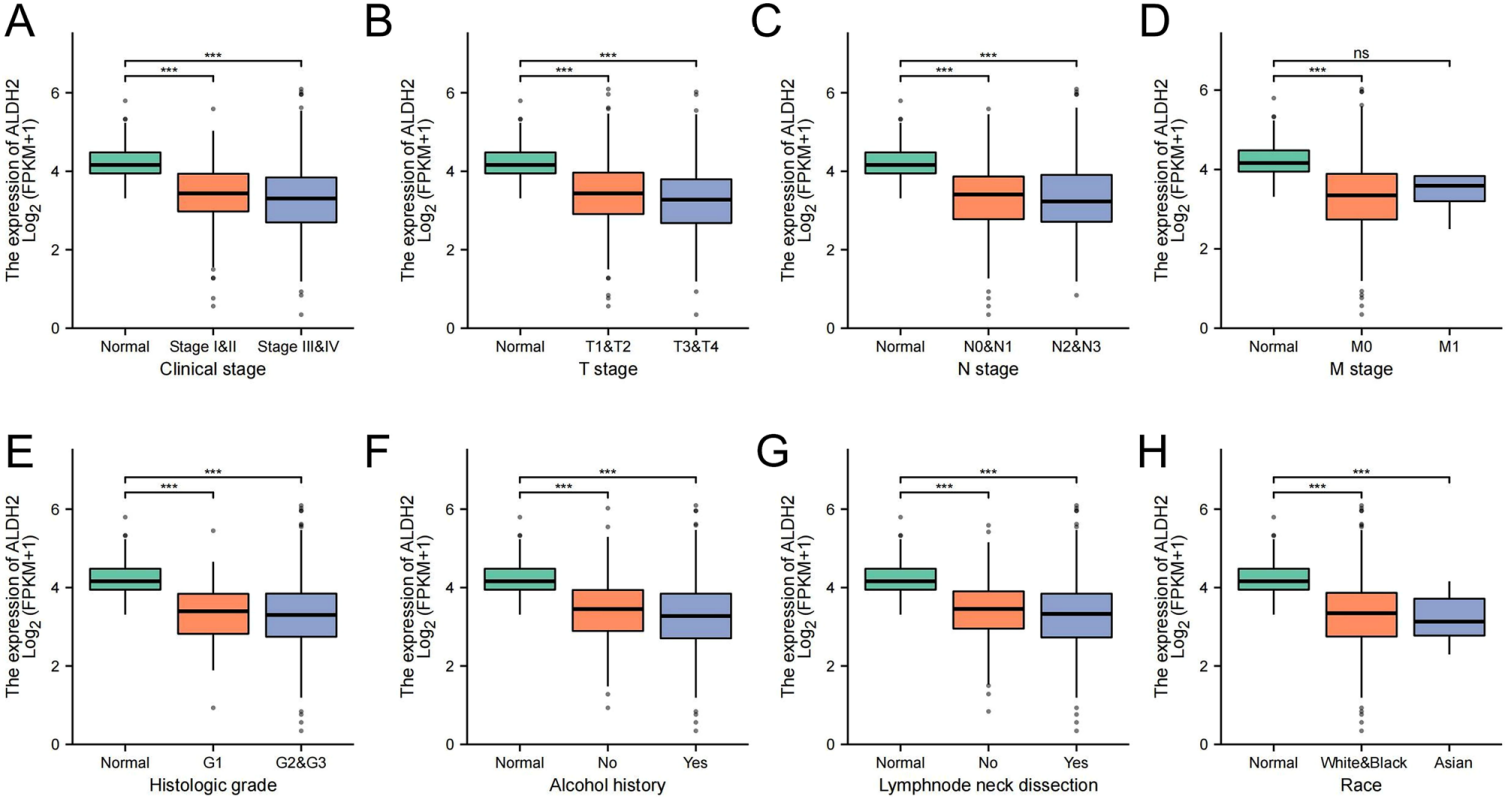
Association between clinicopathological characteristics and ALDH2 expression. (**A**) Clinical stage. (**B**) T stage. (**C**) N stage. (**D**) M stage. (**E**) Histological grade. (**E**) Alcohol history. (**E**) Lymph node dissection. (**E**) Race.

The clinicopathological data of 502 HNSC patients from TCGA were analysed, including clinical stage, age at diagnosis, alcohol history, race, residual tumour, primary tumour, node, and metastasis classification (TNM). As shown in Fig. 3A–H, a decrease in ALDH2 expression was significantly correlated with staging and TNM staging.

Univariate logistic regression confirmed that the expression of ALDH2 was a classified dependent variable with better clinicopathological prognosis (Table 2). Decreased HNSC ALDH2 expression was correlated with T stage (T2 vs. T1, OR = 0.836; T3 vs. T1, OR = 0.485; and T4 vs. T1 OR = 0.643).

**Table 2:** Logistic regression of clinical pathological characteristics and ALDH2 expression.

Clinical Characteristics	Total (N)	OR	95% CI	*P* value
**Clinical stage**				
Stage II vs. Stage I	114	0.736	0.254–1.996	0.555
Stage III vs. Stage I	121	0.519	0.180–1.396	0.202
Stage IV vs. Stage I	291	0.566	0.205–1.452	0.247
**T stage**				
T2 vs. T1	177	0.836	0.379–1.793	0.649
T3 vs. T1	164	0.485	0.218–1.048	0.069
T4 vs. T1	212	0.643	0.295–1.358	0.253
**N stage**				
N1 vs. N0	319	0.881	0.530–1.467	0.624
N2 vs. N0	393	0.647	0.430–0.971	0.036
N3 vs. N0	246	0.133	0.007–0.794	0.064
**M stage**				
M1 vs. M0	477	1.475	0.242–11.273	0.672
**Gender**				
Male vs. Female	502	0.850	0.571–1.262	0.420
**Age**				
> 65 vs. ≤ 65	501	1.041	0.733–1.478	0.822

### Network analysis of the differentially expressed genes correlated with ALDH2 in HNSC

**Figure 4:**
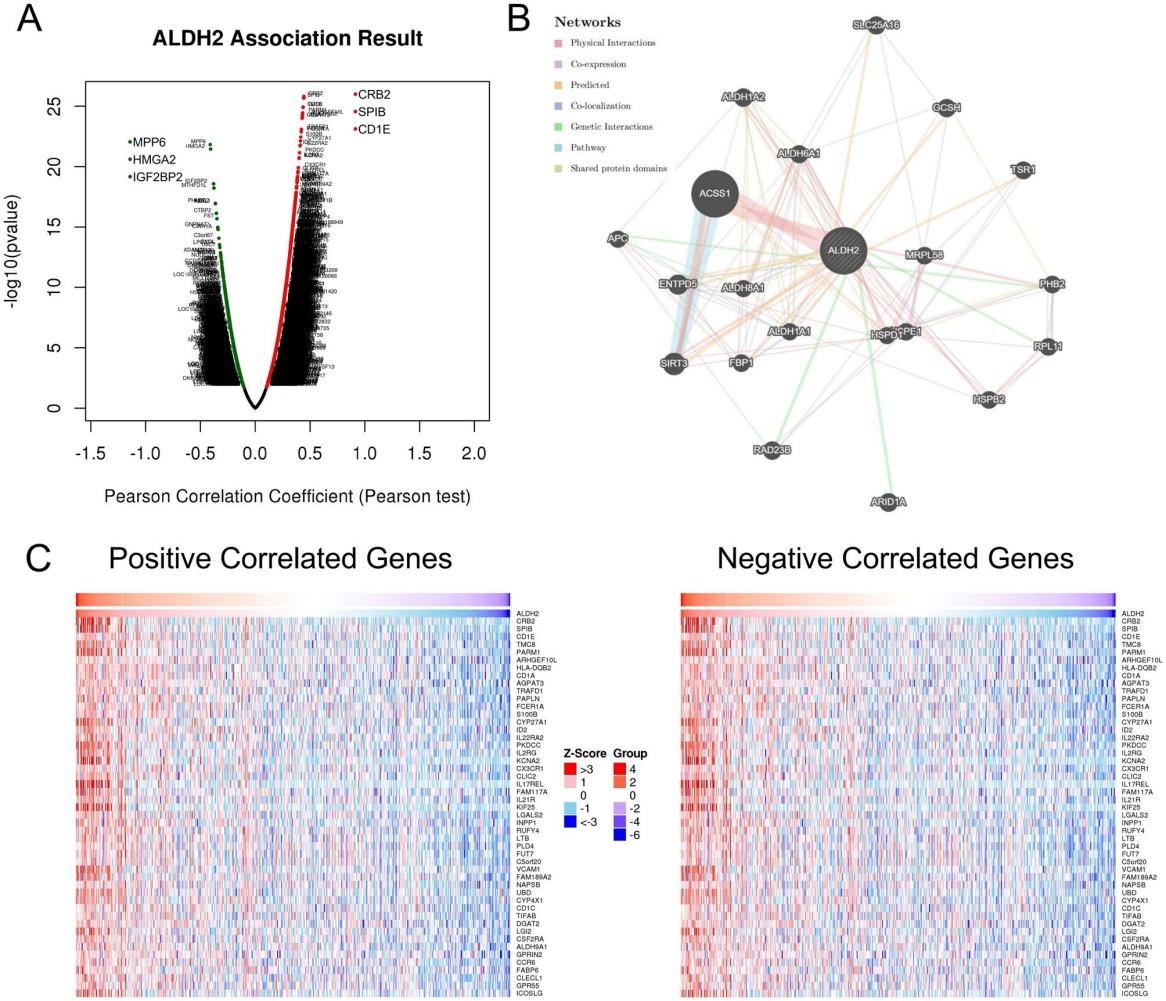
Network analysis of the differentially expressed genes correlated with ALDH2 in HNSC. (**A**) The volcano plot shows the genes that were highly correlated with ALDH2 in HNSC. Green and red dots represent genes that had a significant negative and positive correlation with ALDH2, respectively. (**B**) Main ALDH2 gene cluster in GeneMania analysis results. (**C**) Heatmaps showing the top 50 genes in HNSC that were positively or negatively related to ALDH2 (http://linkedomics.org).

The mRNA sequencing data of 502 HNSC patients from TCGA were analysed by the functional module of LinkedOmics. As shown in the volcano plot (Fig. 4A), red dots indicate a significant positive correlation with the ALDH2 gene, whereas green dots represent a significant negative correlation with the ALDH2 gene (false discovery rate [FDR] < 0.01). The interacting gene network was predicted using GENEMANIA (Fig. 4B). The heatmap shows 50 gene sets that were significantly positively or negatively correlated with ALDH2 (Fig. 4C).

### Function and enrichment analyses

**Figure 5:**
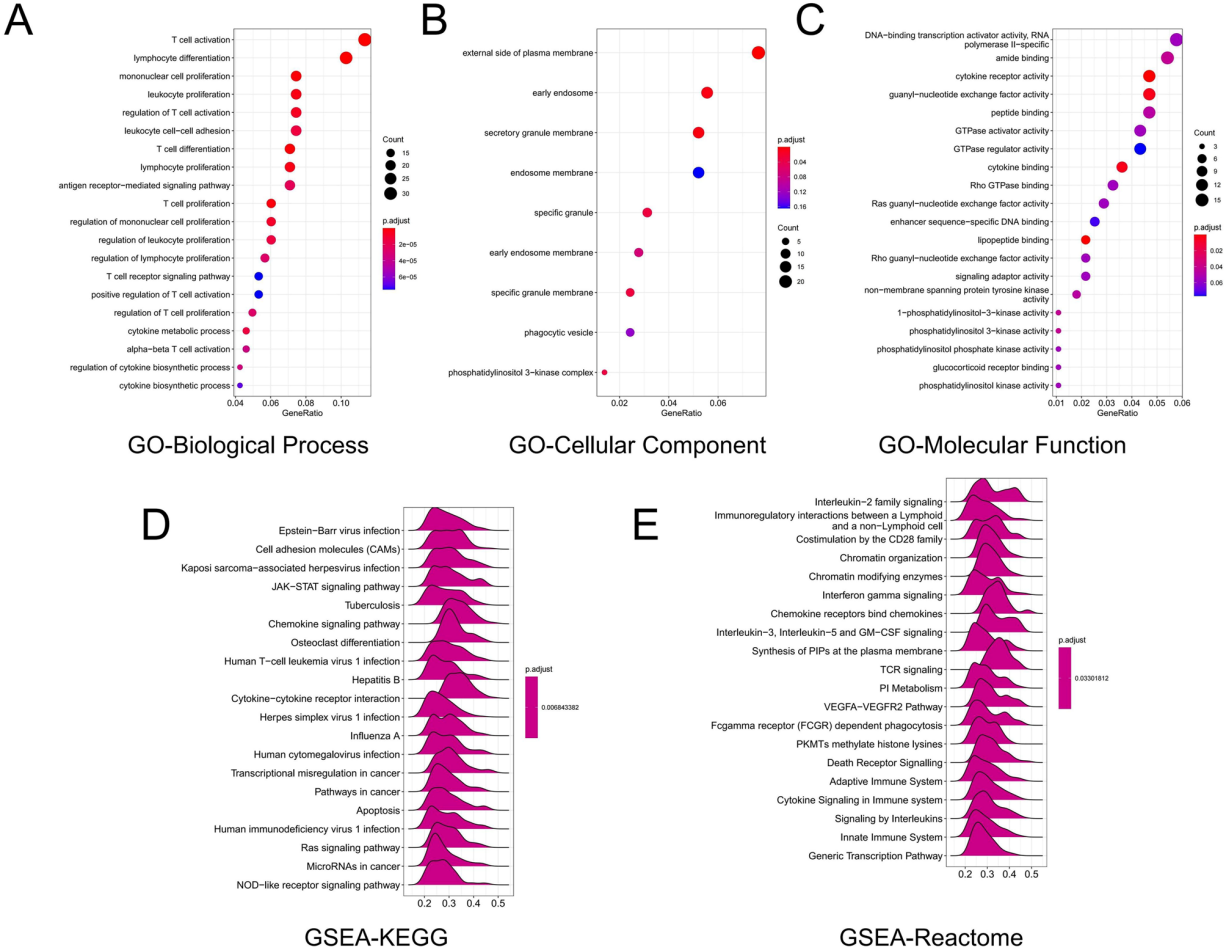
Enrichment analysis of ALDH2 in HNSC (pathview package, version: 1.1.7). (**A**) Significant Gene Ontology terms of the 300 top genes most related to ALDH2 in the biological process category. (**B**) Significant Gene Ontology terms of the 300 top genes most related to ALDH2 in the cell component category. (**C**) Significant Gene Ontology terms of the 300 top genes most related to ALDH2 in the molecular function category. (**D**) Significant gene set enrichment analysis (GSEA) of ALDH2 in KEGG pathways. (**E**) Significant gene set enrichment analysis (GSEA) of ALDH2 in Reactome pathways.

The clusterProfiler R software package was used to analyse highly correlated gene groups to explore possible functional pathways. GO functional enrichment analysis indicated that ALDH2 was correlated primarily with immune cell proliferation-related pathways, including T cell proliferation and regulation of leukocyte proliferation (Fig. 5A–C). GSEA was used to search the Reactome pathway and Kyoto Encyclopaedia of Genes and Genomes (KEGG) databases. The KEGG results indicated that the JAK-STAT signalling pathway, transcriptional misregulation in cancer and microRNAs in cancer were significantly enriched (Fig. 5D). Reactome pathway analysis revealed significant enrichment in the VEGFA-VEGFR2 pathway, death receptor signalling and adaptive immune system pathways (Fig. 5E). These results indicated that ALDH2 expression is correlated with complicated oncogenic pathway hyperactivation in head and neck squamous carcinoma, especially signalling that correlates with cell proliferation and the immune system.

### ALDH2 Expression is Correlated with the Immune Infiltration Level in HNSC

**Figure 6:**
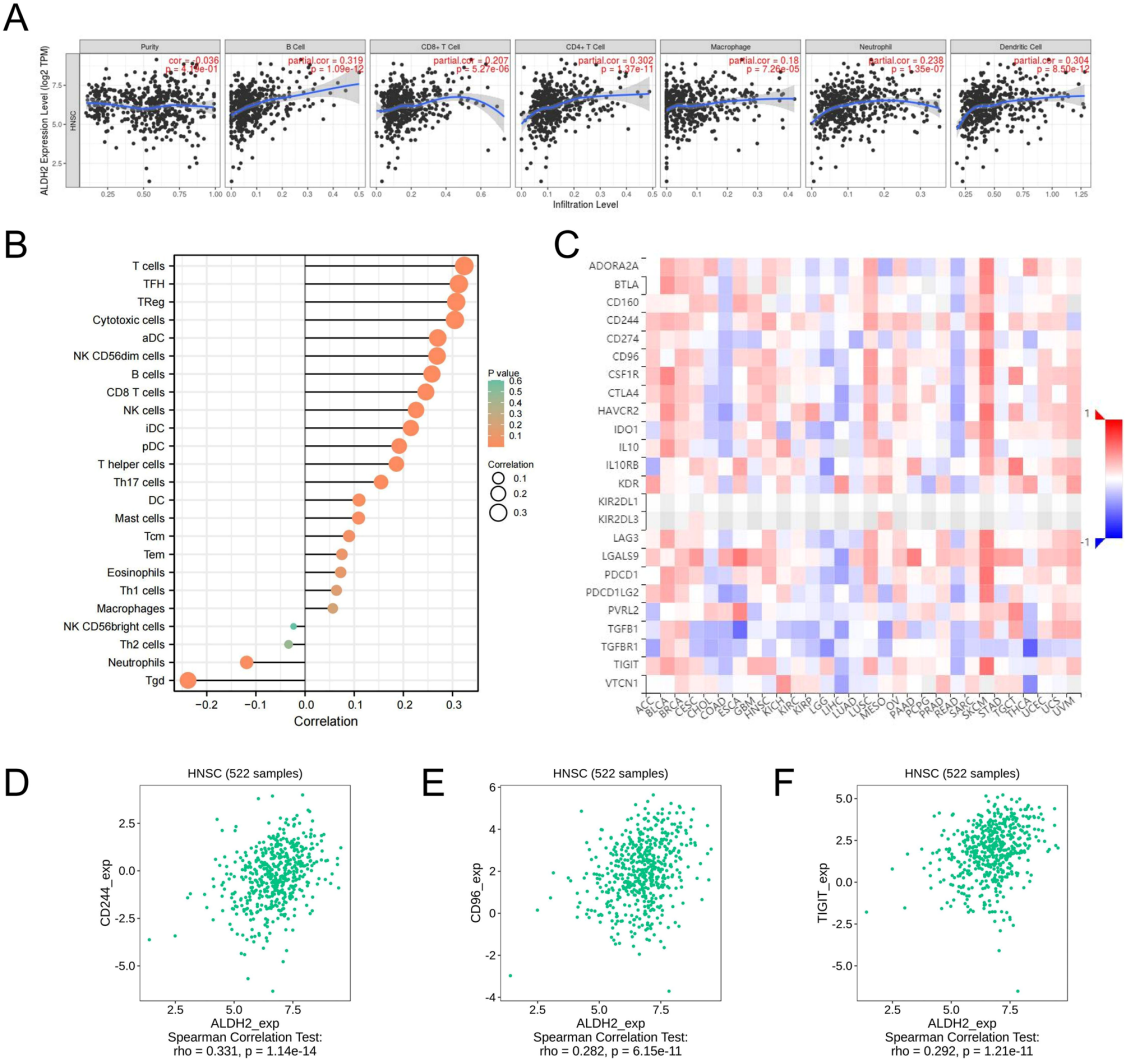
ALDH2 Expression is Correlated with the Level of Immune Infiltration in HNSC. (**A**) Correlation of immune infiltration with ALDH2 expression according to TIMER and analysed by a purity-corrected Spearman’s test. (**B**) Immune infiltration levels of ALDH2 as shown by a lollipop diagram. (**C**) Immune inhibitors that might be regulated by ALDH2 (http://cis.hku.hk/TISIDB). (**D-F**) The expression level of ALDH2 had significant positive correlations with immune inhibitors (CD244, CD96 and TIGIT).

Tumour-infiltrated lymphocytes are independent predictors of the survival and state of cancer lymph nodes^6^. We next explored whether ALDH2 is correlated with the immune infiltration level in head and neck squamous carcinoma. The expression of ALDH2 had significant positive correlations with the infiltration levels of CD8 + T cells (r = 0.207, P = 5.27e-06), CD4 + T cells (r = 0.302, *P* = 1.37e−11), macrophages (r = 0.18, *P* = 7.26e−05), neutrophils (r = 0.238, *P* = 1.35e−07) and DCs (r = 0.304, *P* = 8.50e−12) in HNSCs (Fig. 6A, B). In addition, we investigated immune inhibitors that might be regulated by ALDH2 (Fig. 6C). The expression of ALDH2 also had a significant positive correlation with immune inhibitors of CD244 (r = 0.331, *P* = 1.14e−14), CD96 (r = 0.282, *P* = 6.15e−11) and TIGIT (r = 0.292, *P* = 1.21e−11) in HNSCs (Fig. 6D–F).

### Survival analysis suggests prognostic significance of ALDH2

**Figure 7:**
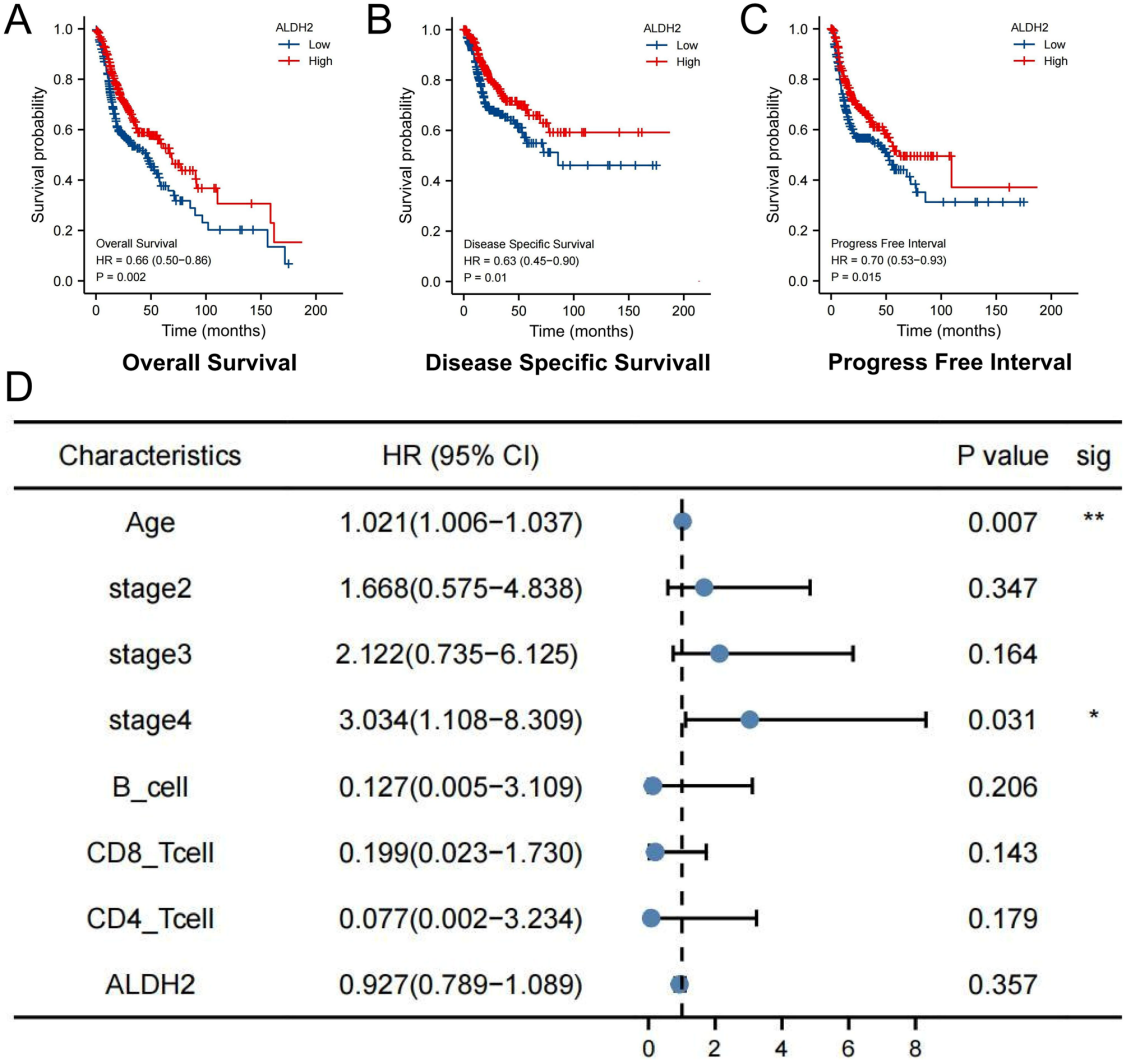
Survival analysis suggests prognostic significance of ALDH2. (**A**) Correlation between ALDH2 expression and the overall survival of HNSC. (**B**) Correlation between ALDH2 expression and the disease-specific survival of HNSC. (**C**) Correlation between ALDH2 expression and the progression-free interval of HNSC. (**D**) Multivariable Cox proportional hazard analysis of ALDH2 expression in HNSC patients.

Disease-specific survival, overall survival and progression-free survival were analysed using the Kaplan–Meier method. Interestingly, high ALDH2 expression correlated with good prognosis in HNSC patients (Fig. 7A–C). A multivariable Cox proportional hazard model is shown in Fig. 7D, which indicated that age (*P* < 0.01) and stage (*P* < 0.05) were important covariates in predicting survival.

**Figure 8:**
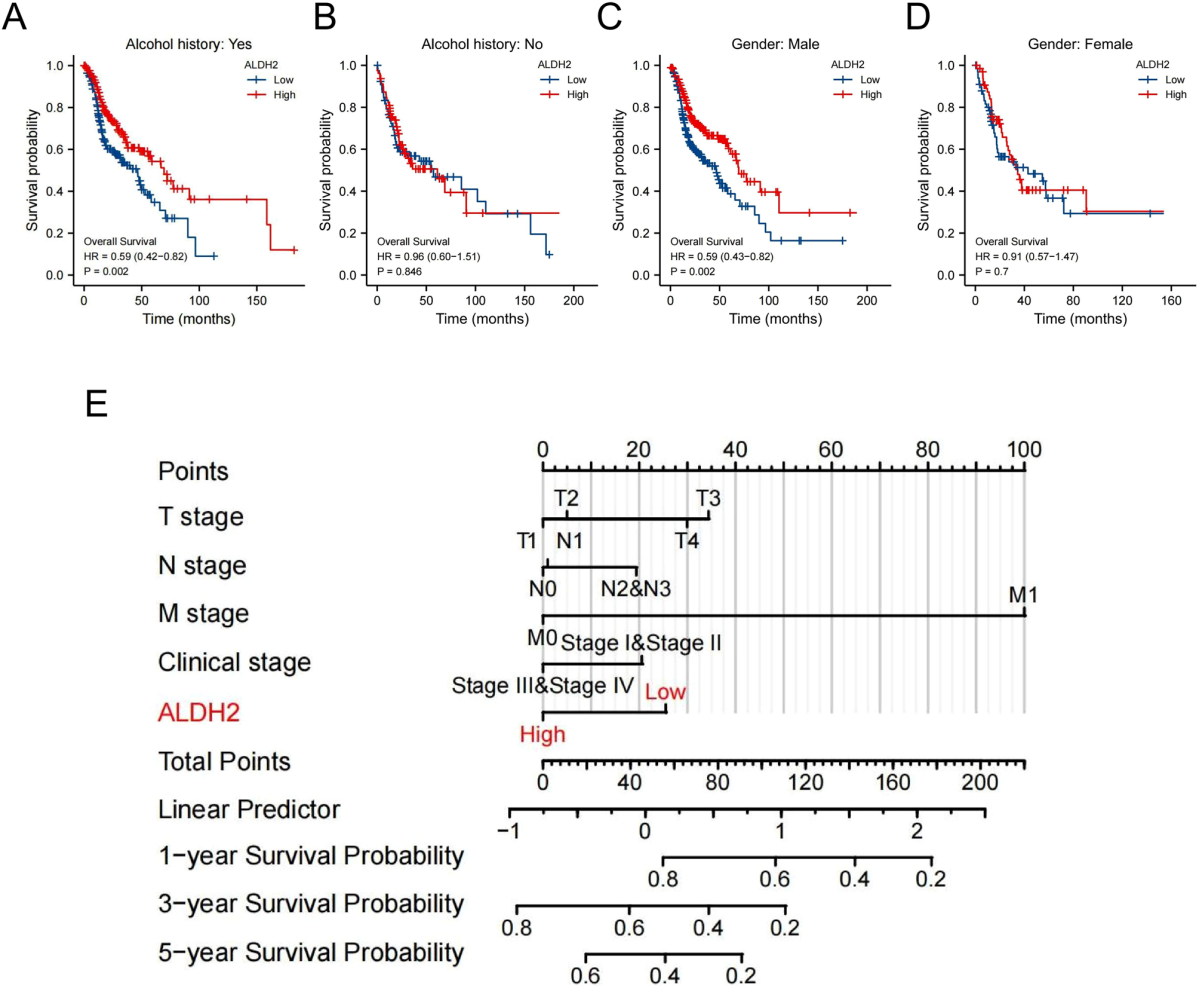
ALDH2 has significant implications for HNSC patients. (**A-D**) ALDH2 expression had different effects on survival based on drinking behaviours and sex. (**E**) Nomograms predicting survival in HNSC patients (rms package-6.2.0).

Surprisingly, our subgroup analysis of overall survival showed a significant difference in overall survival between the alcohol history and sex (Fig. 8A–D), underlining the importance of ALDH2 in predicting the outcomes of male patients who are regular drinkers. Finally, we established a nomogram combining prognostic information from clinicopathologic data and ALDH2 expression to predict the prognosis of HNSC patients (Fig. 8E). The findings indicated that the ALDH2 expression level has important implications for the survival prediction of HNSC patients.

## Discussion

ALDH2, located on chromosome 12q24.12, belongs to the aldehyde dehydrogenase family of proteins^7^. Although previous studies have shown that significant differences in the ALDH2 genotype lead to different prognoses in cancer patients^8,9^, its biological roles and prognostic value in HNSC have rarely been characterized. To our knowledge, this is the first study to assess the influence of alcohol intake combined with ALDH2 expression on clinical survival in HNSC patients. Our findings contribute to existing knowledge, strengthen treatment design and improve the prognostic classification for HNSC patients.

In the present study, we found that ALDH2 was downregulated in HNSC cancer tissues compared to normal tissues and that high ALDH2 expression indicated a good prognosis and was associated with lower tumour stage. The results of univariate and multivariate Cox analyses indicated that ALDH2 might be a prospective independent biomarker for the prognosis of HNSC. We also explored the regulator networks and genes significantly correlated with ALDH2. Finally, a correlation analysis between immune signatures or immune infiltration and ALDH2 was conducted. The results showed that ALDH2 was correlated with most immune marker genes and that T cell infiltration may be an important prognostic factor. The findings of the present study will guide research on HNSC in the future.

To explore the mechanism by which ALDH2 prevents the progression and occurrence of HNSC, DEGs were screened through correlation analysis, and functional annotation and pathway analyses performed. A gene network was established, the functional annotation and pathway analysis of the genes in the main gene clusters were explored. The results suggested that ALDH2 mainly affects the occurrence and progression of HNSC through the JAK-STAT signalling pathway (Fig. 5).

The JAK-STAT pathway is one of the most significant pathways in HNSC. In head and neck carcinoma, STATs are initiated through a variety of signal transduction pathways, including epidermal growth factor receptor (EGFR), erythropoietin receptors and interleukin (IL) receptor pathways^10^. The functional enrichment analysis found that ALDH2 was related to the JAK-STAT pathway, indicating that the possible mechanism of the differential expression and prognostic value of ALDH2 in HNSC is related to the JAK-STAT pathway. A recent study has confirmed the inhibitory effect of aldehyde on the JAK2 signalling pathway through in vitro experiments^11^, further suggesting the possibility that ALDH2 participates in the JAK pathway. However, further experiments are needed to confirm the conclusions of this research. Correlation analysis (Supplemental Fig. 1) indicated that ALDH2 was positively correlated with JAK family genes, thus providing a direction for future research. In the future, in vivo and in vitro experiments should be used to explore the regulatory mechanism of ALDH2 in the JAK/STAT pathway.

GSEA also indicated that the expression of ALDH2 was related to the death receptor signalling pathway. Previous studies have suggested that death receptor signalling is related to antigen-independent drug resistance in leukaemia by inducing CAR-T cell dysfunction^12^. However, it remains to be further explored whether ALDH2 modulates HNSC progression via the death receptor pathway.

Immune cells in the tumour microenvironment are important components that regulate the progression behaviours of tumour cells^13–16^. Another significant feature of this research was the correlation between ALDH2 expression and diverse levels of immune infiltration in HNSC. Our results showed that ALDH2 expression was related to the infiltration level of macrophages, neutrophils, CD8 + T cells, DCs, CD4 + T cells, and B cells in HNSC (Fig. 6A, B). In addition, the correlation between immune inhibitors and ALDH2 expression suggested that ALDH2 regulates tumour immunity in HNSC. These correlations suggested the potential mechanism by which ALDH2 regulates T cell function in HNSC, indicating that ALDH2 plays a key role in the recruitment and regulation of HNSC immune-infiltrating cells. Because ALDH2 is closely related to the immune system, it is worth investigating the role of ALDH2 in cancer immunotherapy. Recently, several researchers have found that ALDH2 mediates the immune evasion induced by alcohol in colorectal cancer by stabilizing the expression of PD-L1^17^. It is also worthwhile to examine whether ALDH2 has this effect in HNSC immunotherapy. Although we used TCGA data to explore the immune infiltration of ALDH2 in HNSC, such statistical inferences may only be suggestive. Future research should involve the collection of large-scale case data for immunological testing to promote the application of ALDH2 in HNSC immunotherapy.

Levels of alcohol drinking are closely related to various types of cancer^18^. ALDH2 dysfunction initiates numerous diseases, such as cardiovascular diseases and cancer^19^. Previous studies have not highlighted the independent prognostic ability of ALDH2, but it was confirmed in our work. Unexpectedly, we found a significant prognostic effect of ALDH2 for HNSC patients based on alcohol consumption and the male sex. Cox analysis suggested that ALDH2 may be a potential independent biomarker for the prognosis of HNSC. In the multivariable Cox proportional hazard model, only the Stage 4 and age subgroups were statistically significant. In the prognostic analysis of subgroups (Supplemental Fig. 2), ALDH2 had a more significant prognostic significance in the advanced groups (i.e., T3, T4, N1, N2, N3, Stage III and Stage IV), suggesting that the prognostic value of ALDH2 in early HNSC is limited. Therefore, when using ALDH2 for prognostic prediction, attention should be given to the priority groups, that is, alcohol consumption, men, and advanced stage.

In summary, medical and biological research on ALDH2 has received increasing attention^7^. Additionally, Alda-1, which restores the activity of the ALDH2*2 enzyme, has the potential to reverse some of the ALDH2*2 population. Therefore, Alda-1 may be used to improve the normal function of ALDH2 and improve the prognosis of HNSC patients^20,21^. This pilot study had several limitations. For example, the present study was a retrospective study. The role and prognosis of the ALDH2 gene in HNSC require a prospective and functional study to provide more accurate information.

## Conclusion

This is the first study describing the correlation between the prognosis of alcohol drinkers and ALDH2 gene expression levels. This research provided comprehensive evidence for the function of ALDH2 in the progression of HNSC and its potential as a prognostic predictor and biotherapy target. These findings can be used to forecast the survival prognosis of patients, especially HNSC patients who are men and alcoholics.

## Materials and methods

### TCGA RNA-sequencing data

In total, 502 HNSC cases and 44 normal samples were included in the gene sequence expression data. Detailed information on the clinicopathological data was downloaded from TCGA data portal (https://www.cancer.gov/). The RNA-Seq gene expression data and clinicopathological data of 502 patients were processed and further analysed (Table 1). This research was conducted under the provisions of the Declaration of Helsinki (revised in 2013).

### Differently expressed genes (DEGs) and gene functional analysis

We performed Pearson correlation analysis to explore the genes significantly correlated with the expression of ALDH2, and the differentially expressed genes (DEGs) were retrieved using the heatmap package of R software. The clinical and mutation information for HNSC patients was obtained from cBioPortal^22^. Gene interactions were predicted using GENEMANIA^23^. LinkedOmics includes multiple sets of data of all 32 cancer types from TCGA, which is an open portal site^24^. By using the “LinkFinder” function of LinkedOmics, we performed the Pearson’s test for statistical analysis of ALDH2 coexpression, and we used a volcano map to display the results. The functional annotations of DEGs were performed by KEGG and Gene Ontology (GO) analyses. Gene signalling pathways were analysed using the clusterProfiler (3.5.1)^25^ and pathview packages (1.1.7)^26^ of R software. All software tools of R langulage (heatmap package-1.0.12, clusterProfiler-3.5.1 and pathview packages-1.1.7, rms package-6.2.0) are free and open source. Immune inhibitors that might be regulated by ALDH2 was explored by using TISIDB^27^ database (http://cis.hku.hk/TISIDB).

### Cox proportional hazard model and nomograms

The Cox proportional hazard model was developed using the TIMER survival module^28^. The covariates consisted of clinical factors (sex, age, tumour stage and ethnicity) and gene expression. The survival model enables researchers to study the clinical relevance of subassemblies from the tumour immune system. The results from TIMER were uploaded to R (version 3.6.3). The meta-analysis and forest plot were generated by the ggplot2 package. Nomograms are widely used to predict the prognosis of cancer patients. In this study, a nomogram was constructed using gene expression and clinical factors^29^. The multivariable model nomograms were generated by the rms package (6.2.0)^30^ in R software (3.6.3).

### Immunohistochemistry and evaluation of immunostaining intensity

ALDH2 expression was analysed using the Human Protein Atlas^31^. Immunostaining was performed using a rabbit anti-ALDH2 antibody (HPA051065). IHC staining was graded as high, medium, low or not detected. The IHC intensity was graded as strong, moderate, weak or negative. The quantity of IHC intensity was graded as > 75%, 75–25%, < 25% and none.

### Statistical analysis

R software (3.6.3) was used for all statistical analyses. The relationship between clinicopathological characteristics was assessed by logistic regression and the Wilcoxon signed rank test. Clinicopathological characteristics associated with overall survival (OS) in HNSC patients from TCGA were analysed by Kaplan–Meier methods and Cox regression. The receiver operating characteristic (ROC) curve was analysed using Wilson’s method. Univariate logistic regression was used to analyse the correlation between clinicopathological characteristics and ALDH2 expression. Univariate and multifactorial Cox analyses were used to assess the effect of ALDH2 expression on survival with other clinicopathological characteristics (e.g., grading, staging, lymph node status, distant metastatic status and age). The ALDH2 expression cut-off value was set as the median, and HNSC patients were divided into two groups.

### Ethical approval

All aspects of the work are the responsibility of the author to ensure that issues related to the accuracy or completeness of any part of the work are properly investigated and resolved. This research was conducted under the provisions of the Declaration of Helsinki (revised in 2013). All data used in this research were publicly available, and approval from the local Ethics Committee was not required.

## Supplementary Information

Supplementary Information 1.

Supplementary Information 2.

## Data Availability

The research data belong to TCGA and are available at https://www.cancer.gov/tcga.
