# Supplementary figures and images for: Exploring ALDH2 expression and immune infiltration in HNSC and its correlation of prognosis with gender or alcohol intake
Source: Sci Rep. 2022 Feb 15;12:2504. doi: 10.1038/s41598-022-06244-1 (PMC8847590; doi:10.1038/s41598-022-06244-1)

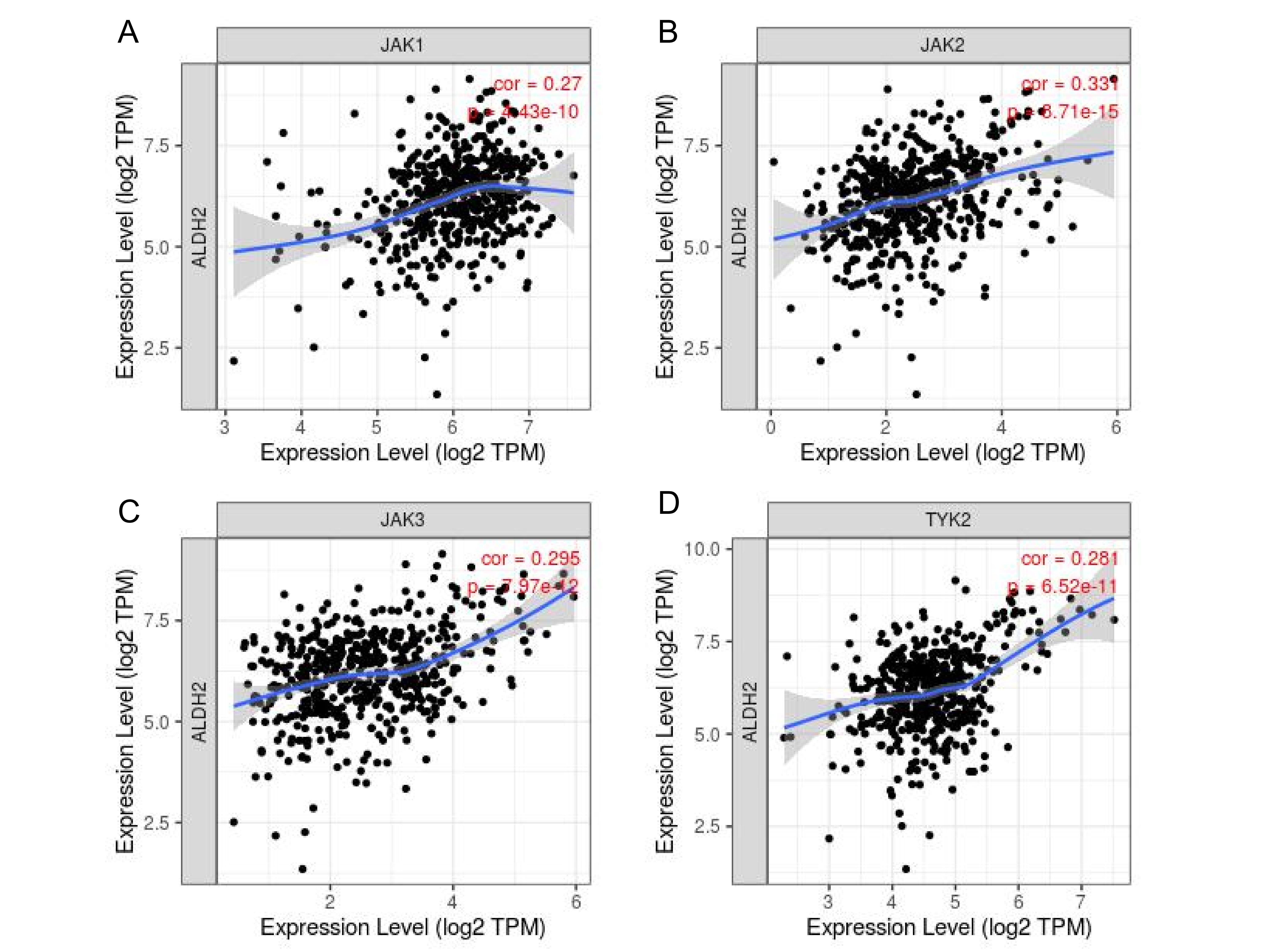

Supplement: Supplementary file 1 — Supplementary Information 1. [file 41598_2022_6244_MOESM1_ESM.jpg]

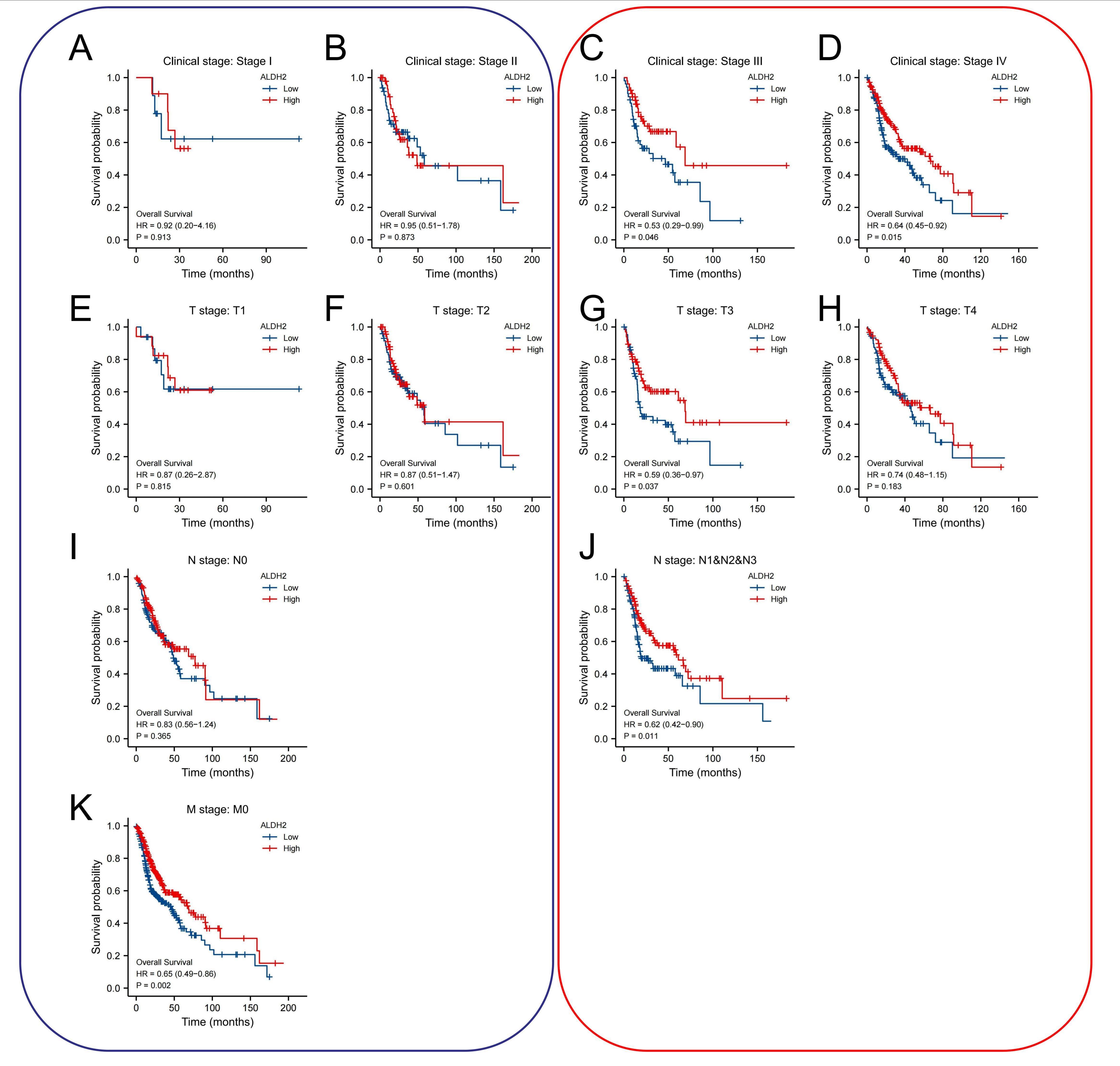

Supplement: Supplementary file 2 — Supplementary Information 2. [file 41598_2022_6244_MOESM2_ESM.jpg]
